# The Lattice Distortion-Induced Ferromagnetism in the Chemical-Bonded MoSe_2_/WSe_2_ at Room Temperature

**DOI:** 10.1186/s11671-022-03692-6

**Published:** 2022-05-27

**Authors:** Shiu-Ming Huang, Pin-Cing Wang, Pin-Cyuan Chen

**Affiliations:** Department of Physics, National Sun Yat-Sen University, 80424 Kaohsiung, Taiwan

**Keywords:** Ferromagnetism, Chemically bonded, Lattice distortion, Two-dimensional transition-metal dichalcogenides

## Abstract

Ferromagnetism to non-ferromagnetism transition is detected in a chemically bonded MoSe$_{2}$/WSe$_{2}$ powder with different thermal annealing temperatures. All samples exhibit ferromagnetism and Raman redshift, except for the 1100 °C thermally annealed sample in which the MoSe$_{2}$ and WSe$_{2}$ are thermally dissociated and geometrically separated. The element analysis reveals no significant element ratio difference and detectable magnetic elements in all samples. These results support that, in contrast to the widely reported structure defect or transition element dopant, the observed ferromagnetism originates from the structure distortion due to the chemical bonding at the interface between MoSe$_{2}$ and WSe$_{2}$.

## Introduction

Spintronics is an approach to manipulating spin polarization and to realizing spin-based functionalities. Dilute magnetic semiconductor (DMS) is one of the promising materials for spintronics applications [1, 2]. The original idea is doping magnetic elements into a semiconductor host, and thus, the material possesses both semiconductor and magnetic behaviors. The DMS has been widely studied in group III-V and II-VI semiconductor-based systems and reveals intrinsic ferromagnetism. However, low Curie temperatures and the intrinsic/extrinsic mechanism disputation limit its application potential [1, 2]. It has been shown that the spin-momentum locking effect is able to induce a non-equilibrium spin accumulation in topological insulators, and this spin accumulation in the topological surface states can be electrically manipulation [3, 4].

The quantum anomalous Hall effect is the quantum Hall effect without an external magnetic field, but instead due to intrinsic ferromagnetism [5]. The quantum anomalous Hall effect is expected to play a role in spintronics. It has been theoretically [5–8] and experimentally demonstrated on the extrinsic ferromagnetic elements in the topological insulator [9–12]. On the hand, it is theoretically and experimentally reported that the structure manipulation could lead to intrinsic ferromagnetism in two-dimensional transition-metal dichalcogenides (2D TMDs) [13–16]. As well as the extrinsic ferromagnetic elements doped, the intrinsic structure manipulation or distortion might lead to ferromagnetism in 2D TMDs. The recent investigation shows that zigzag structure or lattice defect might lead to intrinsic ferromagnetism in 2D TMDs at room temperature [17–27]. Experimental work exhibits that coercivity field, remanence, and saturated magnetization are sensitive to host materials and structure configurations, and it reveals a wide range of performance efficiency. To maximize ferromagnetism, lots of artificial treatments were performed. Most studies mainly focus on the combination of various magnetic elements and host 2D TMDs, as well as the structure and defect treatment methods [17–22]. A recent work exhibits two magnetic elements co-doping treatment in MoS$_{2}$ and WSe$_{2}$ single crystals, and the pinning effect leads to great enhancement of magnetization and coercivity field [28–32]. The ion implantation induces a large number of defects and lattice strains, which serve as pinning centers. The two magnetic elements lead to directional anisotropy coupling strength, and the ferromagnetism is enhanced through the anisotropy distortion. Following this concept, it is interesting to investigate how ferromagnetism would be in a chemical-bonded 2D TMDs.

The chemical-bonded MoSe$_{2}$/WSe$_{2}$ powder (MWS powder) with different thermal annealing temperatures is studied. Ferromagnetism to non-ferromagnetism transition is detected in a chemical-bonded MWS powder with different thermal annealing temperatures. Additionally, a redshift in the Raman peak appears only in systems with “clay-bond” MoSe$_{2}$ and WSe$_{2}$, but not in systems without a geometric connection between MoSe$_{2}$ and WSe$_{2}$. The magnetic field-dependent magnetism shows that a hysteresis loop is observed in systems with chemical bonding at the boundary between MoSe$_{2}$ and WSe$_{2}$, and no hysteresis loops in the system without chemical bonding between MoSe$_{2}$ and WSe$_{2}$. These results support that in contrast to the widely reported structure defect or transition element dopant, the ferromagnetism originates from the structure distortion due to the chemical bonding at the interface between MoSe$_{2}$ and WSe$_{2}$.

## Experimental Method

The chemical-bonded MoSe$_{2}$/WSe$_{2}$ powder (MWS powder) is a commercial product and was purchased from SixCarbon Technology Co. The purchased MWS powder was vacuum-sealed in a glass tube with a pressure of 10$^{-3}$ torr and then, thermally annealed. The MWS powder was heated up to the target temperatures at a rate of 2.7 °C/min, and stayed at the target temperature for 1 hour. After the thermal annealing, it was naturally cooled down to room temperature.

**Fig. 1:**
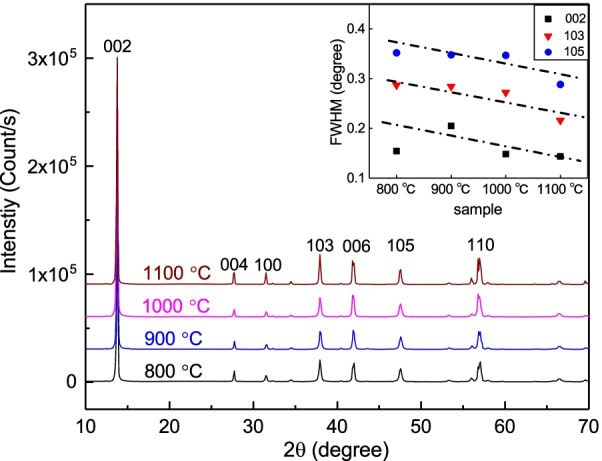
The XRD spectrum of the MoSe$_{2}$/WSe$_{2}$ powder at different thermal annealing temperatures. No obvious peak shift was observed. The inset reveals that the full-width at half height of XRD peaks slightly decrease at higher annealing temperatures

The X-ray diffraction (XRD) was performed in a D2 phaser using the Cu K$\alpha$ radiation with a scan step of 0.1°. Raman spectroscopy was performed in the HORIBA, HR 800 with wavelength 633 nm and scan step of 0.3 cm$^{-1}$. The model of X-ray Photoelectron Spectroscope (XPS) is ULVAC-PHI, PHI 5000 Versa Probe, and the Scanning ESCA Microprobe was used to detect the sample phase composition. The XPS spectra were acquired using a monochromat Al K$\alpha$ source whose energy is about 1486.7 eV. An analyzer acceptance angle of ±8° and take-off angle of 45°, and the pass energy of 15 eV was used for this study. The binding energy scale is calibrated using the ASTM procedure.

**Fig. 2:**
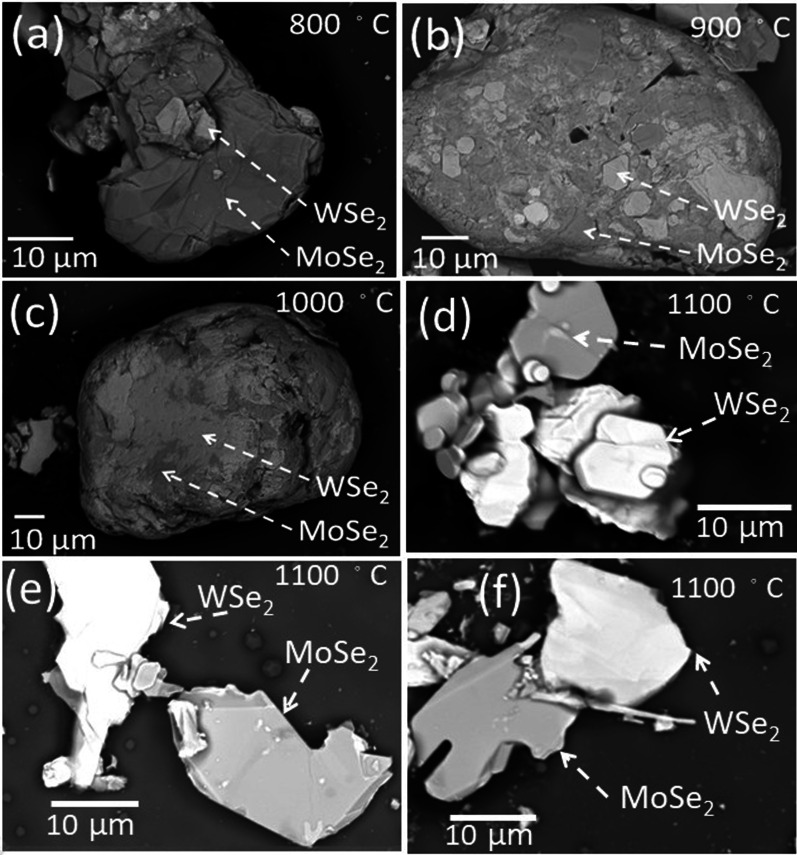
(**a**)–(**f**) show the SEM image of the MoSe$_{2}$/WSe$_{2}$ powder with different thermal annealing temperatures in the backscattering emission image mode. The EPMA result supports that the $\mathrm {W}{:}\mathrm {Se} = 1{:}2$ in the light zone and $\mathrm {Mo}{:}\mathrm {Se} = 1{:}2$ in the dark zone. The MoSe$_{2}$ and WSe$_{2}$ individually locate after 1100° thermal annealed. The others are chemically bonded

The JEOL-6330 Field-Emission SEM backscattering electron image (BEI) mode was used to verify the atomics ratio. Under the BEI mode, one distinguished the MoSe$_{2}$ and WSe$_{2}$ easily by analyzing the secondary electron. The electron beam actually penetrates the sample at about a micrometer depth and scatters back as secondary electrons. The efficiency of backscattering would increase as the atomic number increased. As the result, under BEI mode, the lighter part represents the location of the heavier atom. To remove the possibility of the existence of other atoms, the mapping mode was used. In this mode, SEM can roughly scan a big area (as big as the holder of SEM) on the sample and show the possible existence of atoms in the sample. To exclude the possible impurities and take a higher accuracy atom ratio in our sample, the point mode was used on different shaded areas. Also, in order to get the accurate ratio statistics of the molecular number of MoSe$_{2}$ and WSe$_{2}$, the point mode was applied over hundreds of points. The result implied that the ratio of MoSe$_{2}$ and WSe$_{2}$ is roughly 1.

Magnetism measurements were performed using the standard technique in a SQUID MPMS-3 magnetometer (Quantum Design), and the magnetic field step is 50 Oe.

## Results and Discussion

Figure 1 shows the XRD spectrum of the MWS powder with different thermal annealing temperatures. It reveals sharp peaks and the XRD peak intensity over background noise reaching 440 for (002) peaks in all thermal annealing temperatures. Both MoSe$_{2}$ and WSe$_{2}$ are hexagonal structures, and the XRD peak is consistent with the database of hexagonal structures. No peak intensity and obvious peaks shift were observed in all thermal annealing temperatures, and that indicates that the thermal annealing does not change the crystal structure. Figure 1 inset reveals the full-width at half height (FWHH) of XRD peaks is roughly $0.2 \sim 0.4$. The FWHH slightly decreases as the annealing temperature increases. This supports that the thermal annealing further slightly decreases lattice defects and crystallizes MWS powders.

Figure 2a–f shows the SEM image of MWS in the backscattering emission image (BEI) mode. They are thermally annealed in temperatures from 800 to 1100 °C. Figure 2a–c shows that the MoSe$_{2}$ and WSe$_{2}$ are “clay-bond.” Figure 2d–f shows that the MoSe$_{2}$ and WSe$_{2}$ had thermally dissociated and have rare geometric connection after 1100 °C thermal annealing. The MoSe$_{2}$ and WSe$_{2}$ melting points are roughly 1200 °C which is very close to the highest thermal annealing temperature 1100 °C in this work. That might be the reason why the MoSe$_{2}$ and WSe$_{2}$ are completely dissociated after 1100 °C thermal annealing. The EDS result supports that the $\mathrm {W}{:}\mathrm {Se} = 1{:}2$ in the light zone and $\mathrm {Mo}{:}\mathrm {Se} = 1{:}2$ in the dark zone. Electron probe micro-analyzer (EPMA) was used to determine the material composition ratio. The EPMA supports that the $\mathrm {W}{:}\mathrm {Se} = 1{:}2$ in the light zone, $\mathrm {Mo}{:}\mathrm {Se} = 1{:}2$ in the dark zone, and $\mathrm {MoSe}_{2} {:} \mathrm {WSe}_{2} \approx 1{:}1$. The thermal energy assists the atom in crossing the potential barrier. Atoms prefer to move to the position with lower energy and bond together, and that makes the defect gather together. At higher temperatures, the defects gather together form a bigger defect. As shown in Fig. 2a–c, the morphology exhibits more and bigger “hole” at higher thermal annealing temperatures. These cracks completely separate the MoSe$_{2}$ and WSe$_{2}$ at a critical temperature above 1000 °C. It is reported that thermal annealing induces S vacancies in WS$_{2}$ and MoS$_{2}$. The XPS reveals no obvious Mo, W and Se vacancy difference in the different thermally annealed MWS. Structure vacancy might shift the peak position and suppress the peak intensity. The peak shift and the peak intensity suppression are not observed in Fig. 1, since the thermal annealing-induced element vacancy or structural defect is not an important factor in our MWS.

**Fig. 3:**
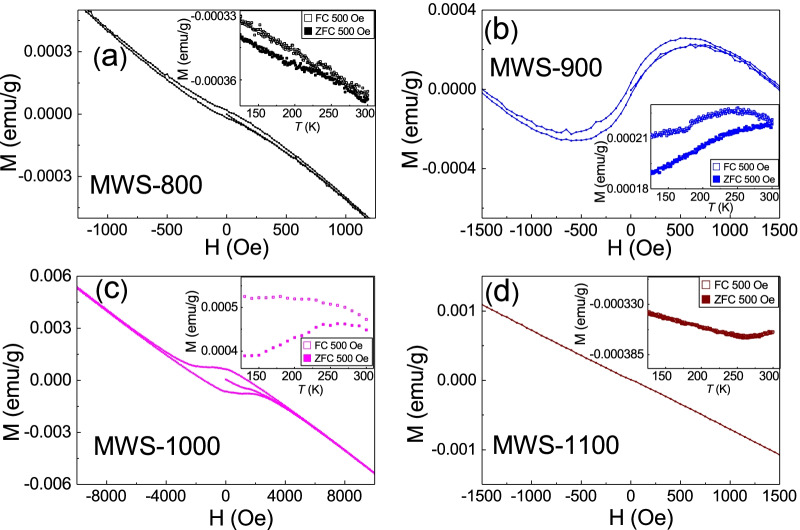
The magnetic field-dependent magnetization. Figure (**a**–**c**) reveals hysteresis loops. Figure **d** shows diamagnetism feature and no hysteresis loops. The inset shows the magnetization of field cool and zero field cool processes. The field cool and zero field cool curve split in all of the samples except for the MWS-1100

**Fig. 4:**
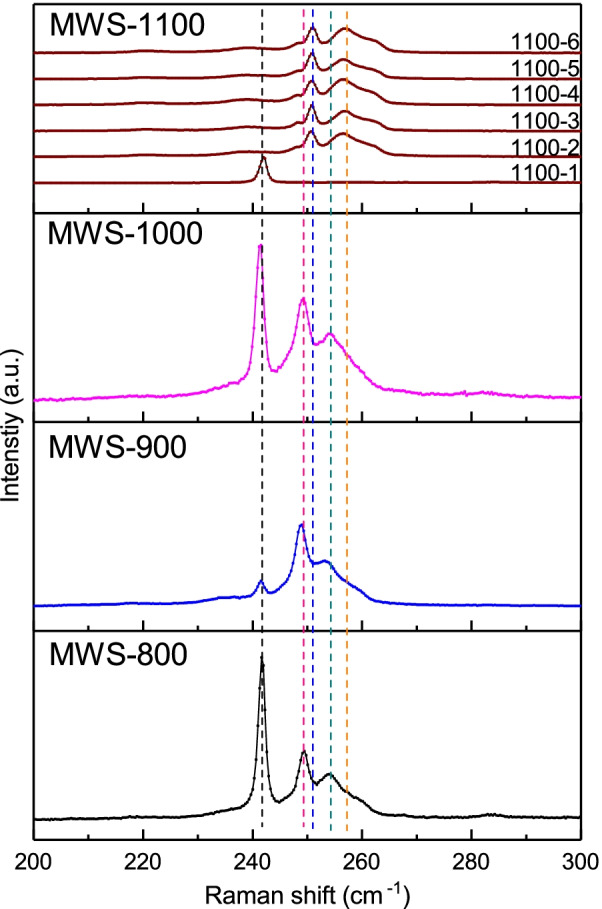
It is 242 cm$^{-1}$ (MoSe$_{2}$, A$_{\mathrm {1g}}$, black dash line), 251 cm$^{-1}$ (WSe$_{2}$, A$_{\mathrm {1g}}$, blue dash line) and 257 cm$^{-1}$ (WSe$_{2}$, 2LA (M), orange dash line) in the Raman spectra. The 249 cm$^{-1}$ (WSe$_{2}$, A$_{\mathrm {1g}}$, red dash line) and 254 cm$^{-1}$ (WSe$_{2}$, 2LA (M), green dash line) are the red shift of the oscillation mode WSe$_{2}$ A$_{\mathrm {1g}}$ and WSe$_{2}$, 2LA (M), respectively. The Raman red shift peak is only observed in the MWS-800, MWS-900 and MWS-1000

Figure 3a–d shows the M-H loop at room temperature, and it exhibits a hysteresis loop in the MWS with 800 °C (MWS-800), 900 °C (MWS-900), and 1000 °C (MWS-1000). It is a ferromagnetism feature. The M-H loop reveals a negative linear diamagnetism in the MWS 1100 °C (MWS-1100). The M-H loop is completely overlapping at a wide range of magnetic fields, and no detectable hysteresis loops are observed in the MWS-1100. To further confirm the existence of the observed ferromagnetism, the field cool (FC) and the zero-field cool (ZFC) processes are performed. As shown in the inset of Fig. 3, the FC and ZFC magnetization splits in the MWS-800, MWS-900, and MWS-1000, and it completely overlapped in the MWS-1100 (Fig. 4). This implies that the ferromagnetism in the MWS-800, MWS-900, and MWS-1000 and is consistent with the M-H loop in Fig. 3.

Moreover, we seek to know the source of the ferromagnetism and the reason why ferromagnetism is not detected in the MWS-1100. It is known that a slight magnetic or transition element dopant might lead to great ferromagnetism in 2D TMDs.

The MWS with different thermal annealing temperatures are from the same raw material and have no difference in treatment processes and equipment. It is expected that the magnetic element concentration would be the same and its contribution to the ferromagnetism would be similar. Figure 3 exhibits that the coercivity field, remanence, and M-H curves are completely different. The MWS-1100 even shows no hysteresis loops. The coercivity fields and magnetization in the MWS-1000 are one-order larger than those in the MWS-800 and MWS-900. On the other hand, the extracted saturated magnetization in our MWS-800 is roughly 0.0004 emu/g (Table 1). This corresponds to 0.4% of magnetic elements (such as Fe, Co, or Ni) contributed to magnetization. This large concentration is within the detectable range in the EPMA, but our experiment reveals no detectable magnetic elements in all MWS. Our EDS analysis also supports that no un-avoided magnetic or transition elements in the MWS.

**Table 1:** List of Raman spectra peak in bulk MoSe$_{2}$ and WSe$_{2}$

Material	MoSe$_{2}$	MWS	WSe$_{2}$	MWS	WSe$_{2}$
mode	A$_{{\boldsymbol{1}{\mathrm {g}}}}$	red shift	A$_{{\boldsymbol{1}{\mathrm {g}}}}$	red shift	2LA(M)
unit	cm$^{\boldsymbol{-1}}$	cm$^{\boldsymbol{-1}}$	cm$^{\boldsymbol{-1}}$	cm$^{\boldsymbol{-1}}$	cm$^{\boldsymbol{-1}}$
800	241.6	249.4	X	254.3	X
900	241.5	249.0	X	253.8	X
1000	241.5	249.3	X	254.8	X
1100-1	241.9	X	X	X	X
1100-2	X	X	250.7	X	256.6
1100-3	X	X	250.7	X	256.9
1100-4	X	X	250.7	X	256.3
1100-5	X	X	250.7	X	256.6
1100-6	X	X	251.0	X	256.9

The ferromagnetism might originate from the lattice defect or zigzag structure [32–42]. Figure 1 shows that the XRD peaks are extremely sharp and the XRD peak signal to background noise ratio and FWHM are almost the same in different MWSs. The structure defect should not lead to the big difference in the observed ferromagnetism features. However, there is ferromagnetism to non-ferromagnetism transition. Moreover, the defect and zigzag should be uniformly distributed in all MoSe$_{2}$ and WSe$_{2}$ blocks in the MWS. The coercivity field is sensitive to host materials and amount of defect. It is reported that the vacancy-induced ferromagnetism coercivity field in WSe$_{2}$ is roughly one order higher than that in MoSe$_{2}$. In the case where the vacancy and defect dominate the observed ferromagnetism in our MWS, one would expect to observe two hysteresis loop steps from individual MoSe$_{2}$ and WSe$_{2}$ contributions. Figure 3a–c shows only one hysteresis loop in all MWSs. On the contrary, it is not reasonable that the defect is completely from specific materials (MoSe$_{2}$ or WSe$_{2}$), so one cannot simply ascribe the observed one-step hysteresis loop to the contribution from specific materials.

The ferromagnetism is studied in the MoS$_{2-x}$Se$_{x}$ crystal, and results reveal that the ferromagnetism is sensitive to the $\mathrm {Se}/\mathrm {S}$ ratio. It exhibits the largest ferromagnetism in the Mo(S$_{0.49}$Se$_{0.51}$)$_{2}$ nanosheet [43]. This supports that similar to the element vacancy or the zigzag edge structure, the element replacement and dislocation might also lead to the ferromagnetism. Furthermore, ferromagnetism and magnetoresistance hysteresis is observed in a molecular-beam epitaxy grown non-magnetic group IV Ge$_{1-x}$Sn$_{x}$ thin film on Ge buffer layer with a high-resistivity Si(001) as substrate. It forms a Ge$_{1-x}$Sn$_{x}$ alloys at the interface between Ge and Ge$_{1-x}$Sn$_{x}$ thin films. The observed ferromagnetism is understood as the inversion symmetry breaking from atomic disordering in the alloy at the interface between Ge and Ge$_{1-x}$Sn$_{x}$ [47]. These experimental results support that the element dislocation might lead to the ferromagnetism, in addition to the structure defect, transition element dopant, or magnetic dopant.

As shown in Fig. 2, the BEI reveals that the MoSe$_{2}$ and WSe$_{2}$ zones are smoothly bonding and might lead to the Mo$_{x}$W$_{1-x}$Se$_{2}$ structure. This chemical bonding at the MoSe$_{2}$ and WSe$_{2}$ interface would lead to slight lattice bonding distortion and that leads to the ferromagnetism. The observed ferromagnetic hysteresis loop might originate from this effect. Theoretical calculation demonstrates that the structure defect would induce ferromagnetism in MoS$_{2}$ monolayer and mentioned the defect plays an important role in the magnetic properties [44]. Experimentally, it is demonstrated the defect would lead to ferromagnetism in various kinds of 2D TMDs [32–42]. The Raman spectrum is a sensitive tool to detect the chemical bonding configuration. To identify the chemical bonding condition between MoSe$_{2}$ and WSe$_{2}$, the Raman spectrum was performed in MWS powders. Figure shows the Raman spectra of MWS. There are three main oscillation modes in the spectra [45]. It is 242 cm$^{-1}$ (MoSe$_{2}$, A$_{\mathrm {1g}}$, black dash line), 251 cm$^{-1}$ (WSe$_{2}$, A$_{\mathrm {1g}}$, blue dash line), and 257 cm$^{-1}$ (WSe$_{2}$, 2LA (M), orange dash line) in the MWS-1100. They are 242 cm$^{-1}$ (MoSe$_{2}$, A$_{\mathrm {1g}}$, black dash line), 249 cm$^{-1}$ (WSe$_{2}$, A$_{\mathrm {1g}}$, red dash line) and 254 cm$^{-1}$ (WSe$_{2}$, 2LA (M), green dash line) in the MWS-800, MWS-900, and MWS-1000. As shown in Fig. 2, the MoSe$_{2}$ and WSe$_{2}$ are completely separated in the MWS-1100, and the MoSe$_{2}$ and WSe$_{2}$ are partially connected in the MWS-800, MWS-900, and MWS-1000. The chemical bonding would directly influence atoms’ oscillation frequencies and that leads to the Raman peak shift. This implies that the 249 cm$^{-1}$ (WSe$_{2}$, A$_{\mathrm {1g}}$, red dash line) and 254 cm$^{-1}$ (WSe$_{2}$, 2LA (M), green dash line) originate from the red shift of the oscillation mode WSe$_{2}$ A$_{\mathrm {1g}}$ and WSe$_{2}$, 2LA (M). The red shift in the Raman peak is only observed in the MWS-800, MWS-900, and MWS-1000. Table lists the observed Raman spectra. The previous work shows that a larger red shift is observed in the Mo$_{x}$W$_{1-x}$Se$_{2}$ with more Mo elements [46]. Upon further examination of the BEI image, it is believed that the Raman red shift should originate from the chemical bonding at the interface of MoSe$_{2}$ and WSe$_{2}$ blocks. This slight atom dislocation would break the lattice symmetry that leads to the structure distortion at the boundary between MoSe$_{2}$ and WSe$_{2}$. This distortion is equivalent to the structure defects, inducing ferromagnetism [47].

## Conclusion

Ferromagnetism to non-ferromagnetism transition is detected in a chemical-bonded MoSe$_{2}$/WSe$_{2}$ powder with different thermal annealing temperatures. The MoSe$_{2}$/WSe$_{2}$ exhibits the ferromagnetism and Raman red shift, except for the 1100 °C thermally annealed sample in which the MoSe$_{2}$ and WSe$_{2}$ are thermally dissociated and geometrically separated. The element analysis reveals no significant element ratio difference and detectable magnetic elements in all samples. Our experimental studies conclude that in contrast with the widely reported structure defect or transition element dopant, the ferromagnetism originates from the structure distortion due to the chemical bonding at the interface between MoSe$_{2}$ and WSe$_{2}$.

## Data Availability

The datasets generated during and/or analyzed during the current study are available from the corresponding authors on reasonable request.

## Abbreviations

DMS: Dilute magnetic semiconductor
XPS: X-ray photoelectron spectroscopy
BEI: Backscattering emission image
EDS: Energy-dispersive X-ray spectroscopy
